# Case Series of an Intraoral Balancing Appliance Therapy on Subjective Symptom Severity and Cervical Spine Alignment

**DOI:** 10.1155/2013/181769

**Published:** 2013-07-02

**Authors:** Young Jun Lee, Joo Kang Lee, Soo Chang Jung, Hwang-woo Lee, Chang Shik Yin, Young Jin Lee

**Affiliations:** ^1^Department of FCST, Graduate School of Integrated Medicine, CHA University, Seoul 135-081, Republic of Korea; ^2^Spine Research Center, Gimcheon University, Gimcheon 740-704, Republic of Korea; ^3^Department of Dentistry, Graduate School of Integrated Medicine, CHA University, Seoul 135-081, Republic of Korea; ^4^Department of Diagnostic Radiology, Graduate School of Integrated Medicine, CHA University, Seoul 135-081, Republic of Korea; ^5^Acupuncture and Meridian Science Research Center, College of Korean Medicine, Kyung Hee University, Seoul 130-701, Republic of Korea; ^6^Graduate School of Integrated Medicine, School of Medicine, CHA University, Seoul 135-081, Republic of Korea

## Abstract

*Objective*. The objective of this study was to investigate the effect of a holistic intraoral appliance (OA) on cervical spine alignment and subjective symptom severity. *Design*. An observational study on case series with holistic OA therapy. *Setting*. An outpatient clinic for holistic temporomandibular joint (TMJ) therapy under the supervision of the Pain Center, CHA Biomedical center, CHA University. *Subjects*. Ambulatory patients presenting with diverse chief complaints in the holistic TMJ clinic. *Main Measures*. Any immediate change in the curvature of cervical spine and the degree of atlantoaxial rotation was investigated in the images of simple X-ray and computed tomography of cervical spine with or without OA. Changes of subjective symptom severity were also analyzed for the holistic OA therapy cases. *Results*. A total of 59 cases were reviewed. Alignment of upper cervical spine rotation showed an immediate improvement (*P* < 0.001). Changes of subjective symptom severity also showed significant improvement (*P* < 0.05). *Conclusion*. These cases revealed rudimentary clinical evidence that holistic OA therapy may be related to an alleviated symptom severity and an improved cervical spinal alignment. These results show that further researches may warrant for the holistic TMJ therapy.

## 1. Introduction

Temporomandibular disorders (TMDs) are not only a potential cause of nondental pain in the orofacial region such as the temporomandibular joint (TMJ) [1], but they may contribute to various symptoms, including neck pain, depression, chronic fatigue, sleep disorders, decreased productivity, and mood disorder [2]. Among these TMD-associated symptoms, coexistence of neck or head pain and TMDs is very common [3]. The close correlation of TMDs with cervical spine disorders has been reported by several researches [4–7]. One study reported that patients who have TMDs report neck symptoms more frequently than patients who do not have TMDs. At the same time, patients who have neck pain report more signs and symptoms of TMD than those who have no neck pain [8]. Cervical spine disorders were reported in 71% of the TMD patients group and only 40% of the non-TMD group [9]. Limitations in the upper cervical region (C0–C3 level) were reported to be present significantly more in the 31 TMD patients than in the 30 controls [10].

Although there has been a debate about the exact role of intraoral appliances (OA) in management of TMDs, OA can be used as one of the important therapeutic modalities for TMD or related disorders [11]. Several studies have shown that functional disturbances in the cervical vertebrae were significantly reduced through acting upon the muscles of the mouth and jaw with OA or other TMD treatments [12, 13], although another study using radiographic and photographic findings reported no significant change in head and neck posture after intraoral splint use [14]. OA has been one of the main modalities of holistic TMJ therapy with a perspective of balance in the nervous system and whole-body structure [15, 16]. This study aimed to investigate an effect of OA on subjective symptom severity and cervical spine alignment in holistic TMJ therapy cases.

## 2. Materials and Methods 

### 2.1. Subjects and Design

This study was designed as a within-subject prepost comparison observational study with one group. Ambulatory patients who newly visited the outpatient clinic of holistic TMJ therapy were prescribed with their first OA, volunteered to participate in this study, provided an informed consent, and were asked to receive radiologic assessment twice: once with OA and once without OA. Patients may be included if they showed somatic dysfunction in the region of TMJ with manifestations of tissue texture abnormality, asymmetry, restriction of motion, or tenderness, or showed positive sign on applied kinesiology-type manual muscle testing in relation to TMJ. Those patients have been considered as possible candidates for holistic OA therapy [15]. Positive sign on manual muscle testing means a response of change in isometric muscle contraction in relation to a touch to the skin of TMJ area. Chief complaint of patients may vary significantly. This study was performed under the supervision of Pain Center, CHA Biomedical center, CHA University, during the period from December 2006 to November 2007 (Table 1). Records were analyzed for 59 outpatient cases (27 females, 32 males). Mean age was 35.2 (SD 13.3). The study protocol was approved by the institutional review board of the CHA university (IRB no. 08-32).

### 2.2. Intervention: Holistic Intraoral Appliance Therapy

**Figure 1:**
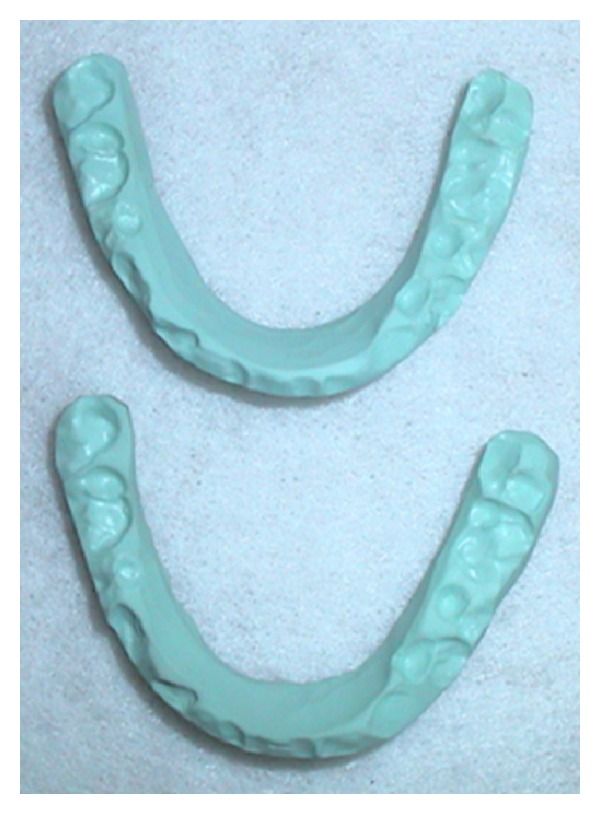
Custom-made intraoral appliance used for holistic temporomandibular therapy.

An OA was individually fabricated and fitted on the spot. OA fabrication was based on the so-called functional cerebrospinal therapy (FCST), originally developed by one of the authors of this paper (Young J. Lee) [15]. As an impression material, vinyl polysilane was used (Exafine putty type, GC Corporation, Japan). On trial-and-error basis, the height and shape of an OA was gradually adjusted for the very individual under examination with continuous monitoring of changes in sign/symptom manifestations, somatic dysfunction, or manual muscle testing. The final height and shape of OA in a given session of treatment was determined on the spot based on the immediate improvement of somatic dysfunction or manual muscle testing (Figure 1) [15]. Thus fabricated OA was intraorally fitted on the spot. The OA thus fabricated was periodically applied by the patient in daily life, as long as symptomatic improvement was maintained. At a follow-up visit, the previous OA might be discarded and a new one be fabricated. 

### 2.3. Radiologic Examination

A CT scan and simple X-ray of cervical spine were taken twice: without application of the OA, followed by with application of the OA. Any significant change in the alignment of the occiput-atlas axis or the cervical spine was explored in relation to the OA. Examination was done within several days (maximum 3 days) after prescribing the first OA for the participant. The patients assumed a supine position facing straight ahead during the examination procedure. The degree of rotational misalignment between the atlas and the axis was measured according to the method of Patijn et al. [17]. Absolute degree of the rotation misalignment between the atlas and the axis was compared in relation to the OA. The patient assumed an erect posture facing straight ahead (paralleling the bite line to the floor) for the lateral X-ray imaging. Degree of lordosis of the cervical spine was measured according to the Cobb technique [18]. The entire CT scan and X-ray imaging were performed by the same radiologist with the same method for all the cases.

### 2.4. Subjective Symptom Severity

The symptom severity data, which were repeatedly documented by the method of a visual analogue scale (VAS) ranging from 0 to 10, where 10 represents unbearable discomfort and 0 represents no discomfort, were additionally reviewed. The VAS severities on the first and last visit were analyzed as a measurement of the clinical picture of the participants without any assumption of direct correlation between cervical alignment and symptom severity. 

### 2.5. Statistical Analysis

Statistical comparisons were performed using SPSS statistical software (version 17 for Windows, SPSS Inc.). Degree of rotational misalignment and severity of subjective symptoms were compared using the Wilcoxon signed-ranks test. Values were presented as mean ± standard deviation with a significance level of 0.05.

## 3. Results

### 3.1. Immediate Changes in Rotational Misalignment Following Use of the Intraoral Appliance

**Figure 2:**
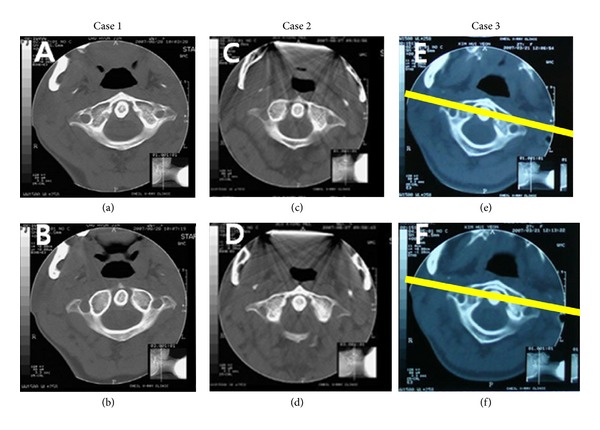
Exemplary CT image of 3 patients. Comparison shows an immediate change in the degree of rotational misalignment in the atlas while wearing the oral appliance. (a), (c), and (e) CT image while not wearing the oral appliance. (b), (d), and (f) CT image while wearing the oral appliance. Lines drawn over figures (e) and (f) indicate ameasuring method of atlas rotation angle with a line passing the anterior border of foramen transversarium of the atlas.

Rotational misalignment in upper cervical spine showed immediate change in 72.4% of the patients. Predominant change was decreased in rotational misalignment after OA's use (*P* < 0.01). When grouped according to type of the change, 62.1% of the patients showed a 2.8-degree decrease (Figure 2), and 10.3% of the patients showed a 2.7-degree increase (Table 2). 

### 3.2. Immediate Changes in Cervical Lordosis Following Use of the Intraoral Appliance

Degree of cervical lordosis showed immediate change in 80.0% of the patients. When grouped according to type of the change, 38.2% of the patients showed a 5.1-degree decrease, and 41.8% of the patients showed a 5.5-degree increase (Table 3). 

### 3.3. Changes in the Severity of Subjective Symptoms

**Figure 3:**
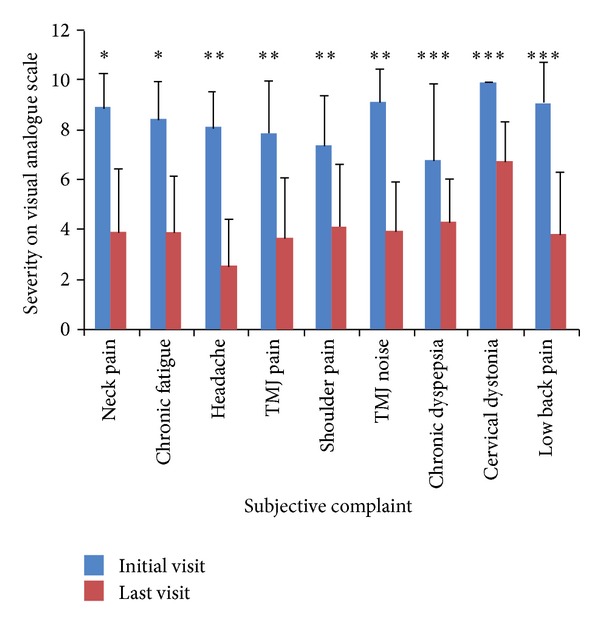
Severity of subjective symptoms compared between initial and last visits. TMJ: temporomandibular joint. *P < 0.05, **P < 0.01, ***P < 0.001 by Wilcoxon signed-rank test.

The patients showed improvement in the severity of subjective symptoms such as neck pain and chronic fatigue (Table 4, Figure 3) after a mean treatment period of 1.5 months with 20.7 treatments (Table 1).

## 4. Discussion

In this study, we found that immediate change in cervical alignment seems to be related with an OA therapy: the degree of rotational misalignment between the atlas and axis was reduced. In addition to that, the participants showed an improvement in subjective symptom severities after 1.5 months, even though this change may not be associated with the OA therapy. Wearing an intraoral appliance seemed to accompany reduced degree of cervical lordosis in those with greater-than-average degrees of lordosis or increased degree of cervical lordosis in those with lesser-than-average degrees of lordosis, coming closer to the average degree of lordosis. However, the average degree of lordosis was not different between the two conditions. We cannot exclude the possibility that these changes in cervical lordosis may just reflect a random variation or the statistical phenomenon of regression to the mean. 

Positive relationship between TMD and cervical spine was reported in previous reports [4, 6]. Although the relationship between TMD and head and neck posture or cervical spine alignment has not been shown clearly [5, 19], clinical or muscular aspects of such relationship may not be so ambiguous, considering increased muscular fatigability of cervical extensor muscles in TMD patients [20] and close correlation between TMD and spinal pain in clinical practice [7]. Increased or decreased lordosis of cervical alignment may be related to increased stress or vulnerability to stress in cervical spine [21–23]. In this study, we found that wearing the intraoral balancing appliance appeared to be related to such change in cervical alignment that increased or decreased cervical lordosis turning into a close-to-average angle of  lordosis. This may be related to decrease in stress or vulnerability to stress of cervical spine. It is suggested that the change into more close-to-average lordosis from decreased lordosis, rather than from increased lordosis, may be more closely related to stress reduction considering that flexion deformity and decreased lordotic curve is a well-described type of aberration in cervical curvature [23].

The close correlation of TMD with cervical spine disorders has been reported previously, and there are several explanations in terms of biomechanical, neuroanatomical, and neurophysiological aspects for these close relations [24]. Neurophysiologically, there is convergence and central excitatory connection between the trigeminal nerve and the trigeminocervical nucleus. For example, afferent input from neck muscles and the cervical spine results in masticatory muscle contraction through the excitation of motor neurons in the trigeminal nerve [25, 26]. Experimental trapezius muscle pain was observed to spread most often to the infraauricular zone and was also accompanied by a temporary reduction of mouth opening [27]. While sensory information from trigeminal primary afferent neurons descends down to C2-C3 and even C6 [28–30], experimentation has shown that electrical stimulation of the infraorbital nerve, a branch of the trigeminal nerve, activates interneurons in the ventral horns of C1 to C4, readily exciting neck muscle motoneurons [31]. In the case of TMD, transmission of nociception from the TMJ by means of the auriculotemporal nerve, the posterior trunk of the mandibular nerve, to the spinal tract of the trigeminal nerve, the trigeminocervical nucleus, and the motoneurons of the cervical spine is the one mechanism through which TMD causes cervical pain and muscle tension changes [32]. Biomechanically, the temporomandibular system and cervical spine behave as one functional unit. Thus, not only could changes in head posture result in cervical spine dysfunction, but cervical spine movement could also result in changes in TMJ movement [33]. With forward head posture, the posterior cervical muscles are shortened isometrically, while the anterior submandibular muscles are stretched to cause retrusive forces on the mandible [34]. Increase in the vertical dimension of the resting mandible was reported to occur with an increase in the angle of habitual head posture and a decrease in the retrusion of the mandible [35]. Simultaneous functional movements in the temporomandibular, atlantooccipital, and cervical spine joints were also reported [36]. Several studies have shown that functional disturbances in the cervical vertebra were significantly reduced by acting upon the muscles of the mouth and jaw as a result of OA or other TMD treatments [12, 13]. The successful correction of unbalanced TMJ position is suggested to be dependent on the health of the cervical and other spine musculature as well as the head position [37, 38]. And OA can possibly reduce the loading inside the TMJ by reducing the intensity of muscle activity or shifting the condylar loading area [39].

This study adopted a CT scanning and simple X-ray imaging for analysis of the cervical spine alignment. For analysis of the positioning of the cervical spinal curve in individuals with TMD, many studies have evaluated cervical spine alignment using cephalometric X-rays. However, evaluation based upon cephalometric X-rays has some limitations, such as artificial positioning. The lateral cervical spine X-ray is also difficult to interpret due to head tilt. In the present study, X-rays were taken with individuals in a natural standing position, with no modifications of the cervical curve. In spite of the reported interexaminer and intraexaminer reliability [40, 41], there exist possible limitations of plain radiography [42–44], which can be overcome using CT assessment. An axial CT through the upper cervical spine demonstrates the rotated position of the atlas on the axis and associated forward or backward displacement of the atlas [45].

There has been debate about the exact role of OA in the management of TMDs, although many types of OA have been used for over 50 years to treat TMDs. In the last 20 years, changes in the pathophysiology of TMDs from simple mechanical and structural concepts to complex neurophysiologic concepts have forced a reconsideration of traditional idea about using OA [46]. But the OA may still be a valuable adjuvant modality in the management of TMDs [11]. There are various OA designs, such as the flat plane stabilization appliance (Michigan splint) [47], the traditional anterior bite plane appliance, the minianterior appliances [48], the anterior repositioning appliance [49], the neuromuscular appliances [50], the posterior bite plane appliances [51], and the hydrostatic appliance [52].

The indication of an OA therapy in this study was not that of the conventional TMD criteria. The indication includes any of the somatic dysfunction signs or positive applied kinesiology-style manual muscle testing, which may actually be said as including even any functional (nonpathologic) imbalance in the TMJ and nearby muscles or static posture or joint movement trajectory. We may not tell that the participants in this study belong to TMD. However, the objective of this study was to explore any relationship between OA therapy and cervical alignment, not the effect on TMD. And we tried to make an approach to OA therapy from perspectives of complementary and alternative medicine rather than the more strict conventional TMD concept or OA therapy. So, we adopted a new type of OA therapy that is developed in the Republic of Korea and is applied for diverse chief complaints with a functional imbalance in the TMJ region from perspectives of balance enhancing in the nervous system [15, 16, 53]. The design of this OA reflects mixed concepts of the flat plane stabilization appliance and neuromuscular appliances.

This study is an observational study on case series without control group. This limitation prevents definite comparison or inference. Further investigation adopting controlled study and more rigid design is warranted. This study only evaluated the immediate change of cervical alignment in relation to an application of OA in one group. So we cannot state definitely any correlation between such a change of cervical alignment and the clinical symptom change. However, the immediate cervical alignment change observed in this study may warrant further investigation into the correlations between the TMJ, OA, subjective symptoms, and cervical alignment. Future studies may also be needed to investigate the dynamic relationship between the cervical spine and TMDs [53]. In order to clarify the dynamic relationship between the movements of the cervical spine and neck muscles and the mandibular opening or closing and masticatory muscles, the complex dynamics of TMJ and the cervical spine need to be analyzed through the use of reliable clinical instrumentation. Through such studies, clinical application of OA therapies will be developed as a therapy with an evidence base.

This kind of holistic TMJ balancing approach is based on Yin-yang's balance concept as an application of meridian balance concept [15]. It was originally developed in Korean medicine in the 1990s [54]. TMJ posture is adjusted with intraoral TMJ balancing appliance that was tailor made based on verification using such functional assessments as palpation on meridian-muscular system, applied kinesiology, and functional neurological examination [15]. Postural training of TMJ using intraoral TMJ balancing appliance may lead to positive whole-body response. Unlike previous reports on episodic cases treated with this holistic approach [55], this study analyzed cases series in which diverse chief complaints are distributed from pain to dyspepsia with additional analysis on possible mechanism of this holistic approach, a close linkage between TMJ postural intervention and change in upper cervical alignment. However, it is unclear if this treatment keeps prolonged effects. Further studies are warranted to convince the effect of intraoral appliance for therapeutics.

In conclusion, this study revealed a significant correlation between OA in holistic TMJ therapy and an immediate change of cervical spine alignment and symptom severity.

## Figures and Tables

**Table 1:** General characteristics of the patients.

	No. (%)
Gender	
Male	32 (54.2%)
Female	27 (45.8%)
Chief complaints	
Pain disorder	33 (55.9%)
Temporomandibular joint pain	9 (15.3%)
Neck pain	8 (13.6%)
Headache	6 (10.2%)
Others	10 (16.9%)
Idiopathic torticollis	9 (15.3%)
Others	17 (28.8%)
Duration of symptoms (years)	
0–2	20 (33.9%)
2–4	15 (25.4%)
4–10	12 (20.3%)
10–16	8 (13.6%)
16–22	2 (3.4%)
22–38	2 (3.4%)
Outpatient treatment	
Period (months)*	1.5 ± 1.7 (0.1–8.1)
No. of treatments*	20.7 ± 33.4 (3–229)

__*Data are presented as mean ± SD (range).

**Table 2:** Immediate change in degree of rotational misalignment between C1 and C2 vertebrae on a computed tomography image.

	No. (%)*	Degree of the rotational misalignment		Immediate change
		Without an intraoral appliance	With an intraoral appliance	
Groups based on type of the change				
Decrease	36 (62.1)	6.1 ± 5.0	3.3 ± 4.1	−2.8 ± 2.0***
No change	16 (27.6)	2.4 ± 2.2	2.4 ± 2.2	0
Increase	6 (10.3)	2.7 ± 2.1	5.3 ± 3.5	2.7 ± 2.7**

Total	58 (100.0)	4.7 ± 4.5	3.3 ± 3.6	−1.4 ± 2.5***

*There were missing data in one case. Values are presented as number (%) or mean ± SD.

**P < 0.05,  ***P < 0.001 by Wilcoxon signed-rank test.

**Table 3:** Immediate change in degree of cervical lordosis calculated by Cobb technique on a simple X-ray image.

	No. (%)*	Mean ± SD		
		Without an intraoral appliance	With an intraoral appliance	Degree of change
Groups based on the type of the change				
Decrease	21 (38.2)	45.3 ± 11.4	40.5 ± 11.0	−5.1 ± 3.7**
No change	11 (20.0)	41.6 ± 3.32	41.6 ± 3.3	0
Increase	23 (41.8)	35.8 ± 10.4	41.4 ± 11.4	5.5 ± 4.7**

Total	55 (100.0)	40.6 ± 12.0	41.1 ± 11.4	0.4 ± 6.1

*There were missing data in four cases. Values are presented as number (%) or mean ± SD.

**P < 0.001 by Wilcoxon signed-rank test.

**Table 4:** Frequent subjective symptom groups and treatment sessions of each group.

Subjective symptoms	No. (%)*	Treatment sessions
Neck pain	39 (70.9)	18.4 ± 20.5
Chronic fatigue	17 (30.9)	21.6 ± 19.3
Headache	14 (25.5)	16.4 ± 23.7
Temporomandibular joint pain	13 (23.6)	15.0 ± 14.3
Shoulder pain	11 (20.0)	6.9 ± 3.6
Temporomandibular joint noise	11 (20.0)	30.8 ± 66.2
Chronic dyspepsia	8 (14.5)	19.3 ± 17.5
Cervical dystonia	8 (14.5)	51.8 ± 75.2
Low back pain	8 (14.5)	19.3 ± 20.1

*There were missing data in four cases. Values are presented as number (%) or mean ± SD.

## References

[1] Okeson JP (1996). *The American Academy of Orofascial Pain: Guidelines for Assessment, Diagnosis, and Management*.

[2] Auvenshine RC (2000). Acute vs. chronic pain. *Texas Dental Journal*.

[3] Cacchiotti DA, Plesh O, Bianchi P, McNeill C (1991). Signs and symptoms in samples with and without temporomandibular disorders. *Journal of Craniomandibular Disorders*.

[4] la Touche R, Fernández-de-las-Peñas C, Fernández-Carnero J (2009). The effects of manual therapy and exercise directed at the cervical spine on pain and pressure pain sensitivity in patients with myofascial temporomandibular disorders. *Journal of Oral Rehabilitation*.

[5] de Farias Neto JP, de Santana JM, de Santana-Filho VJ, Quintans-Junior LJ, de Lima Ferreira AP, Bonjardim LR (2010). Radiographic measurement of the cervical spine in patients with temporomandibular dysfunction. *Archives of Oral Biology*.

[6] Olivo SA, Fuentes J, Major PW, Warren S, Thie NMR, Magee DJ (2010). The association between neck disability and jaw disability. *Journal of Oral Rehabilitation*.

[7] Walczyńska-Dragon K, Baron S (2011). The biomechanical and functional relationship between temporomandibular dysfunction and cervical spine pain. *Acta of Bioengineering and Biomechanics*.

[8] de Wijer A, de Leeuw JRJ, Steenks MH, Bosman F (1996). Temporomandibular and cervical spine disorders: self-reported signs and symptoms. *Spine*.

[9] Alanen PJ, Kirveskari PK (1984). Occupational cervicobrachial disorder and temporomandibular joint dysfunction. *Cranio*.

[10] de Laat A, Meuleman H, Stevens A, Verbeke G (1998). Correlation between cervical spine and temporomandibular disorders. *Clinical Oral Investigations*.

[11] Dao TTT, Lavigne GJ (1998). Oral splints: the crutches for temporomandibular disorders and bruxism. *Critical Reviews in Oral Biology and Medicine*.

[12] Cane L, Schieroni MP, Ribero G, Ferrero M, Carossa S (1997). Effectiveness of the Michigan splint in reducing functional cervical disturbances: a preliminary study. *Cranio*.

[13] Vernon LF, Ehrenfeld DC (1982). Treatment of temporomandibular joint syndrome for relief of cervical spine pain: case report. *Journal of Manipulative and Physiological Therapeutics*.

[14] Root GR, Kraus SL, Razook SJ, Samson GS (1987). Effect of an intraoral splint on head and neck posture. *The Journal of Prosthetic Dentistry*.

[15] Yin CS, Koh HG, Lee YJ, Chun SI, Lee YJ (2005). Functional Cerebrospinal Therapy (FCST), a new physiologic therapeutics developed as meridian yin-yang balance approach. *Korean Journal of Acupuncture*.

[16] Yin CS, Lee YJ, Lee YJ (2007). Neurological influences of the temporomandibular joint. *Journal of Bodywork and Movement Therapies*.

[17] Patijn J, Wilmink J, ter Linden FHJ, Kingma H (2001). CT study of craniovertebral rotation in whiplash injury. *European Spine Journal*.

[18] Malfair D, Flemming AK, Dvorak MF (2010). Radiographic evaluation of scoliosis: review. *American Journal of Roentgenology*.

[19] Armijo-Olivo S, Rappoport K, Fuentes J (2011). Head and cervical posture in patients with temporomandibular disorders. *Journal of Orofacial Pain*.

[20] Armijo-Olivo S, Silvestre RA, Fuentes JP (2012). Patients with temporomandibular disorders have increased fatigability of the cervical extensor muscles. *Clinical Journal of Pain*.

[21] Korovessis P, Koureas G, Zacharatos S, Papazisis Z (2005). Backpacks, back pain, sagittal spinal curves and trunk alignment in adolescents: a logistic and multinomial logistic analysis. *Spine*.

[22] Wei W, Liao S, Shi S, Fei J, Wang Y, Chen C (2013). Straightened cervical lordosis causes stress concentration: a finite element model study. *Australasian Physical & Engineering Sciences in Medicine*.

[23] Öktenoğlu T, Özer AF, Ferrara LA, Andalkar N, Sarioğlu AC, Benzel EC (2001). Effects of cervical spine posture on axial load bearing ability: a biomechanical study. *Journal of Neurosurgery*.

[24] de Laat A (1987). Reflexes elicitable in jaw muscles and their role during jaw function and dysfunction: a review of the literature—part II: central connections of orofacial afferent fibers. *Cranio*.

[25] Hu JW, Yu X-M, Vernon H, Sessle BJ (1993). Excitatory effects on neck and jaw muscle activity of inflammatory irritant applied to cervical paraspinal tissues. *Pain*.

[26] Svensson P, Arendt-Nielsen L, Houe L (1998). Muscle pain modulates mastication: an experimental study in humans. *Journal of Orofacial Pain*.

[27] Komiyama O, Arai M, Kawara M, Kobayashi K, de Laat A (2005). Pain patterns and mandibular dysfunction following experimental trapezius muscle pain. *Journal of Orofacial Pain*.

[28] Bogduk N (1992). The anatomical basis for cervicogenic headache. *Journal of Manipulative and Physiological Therapeutics*.

[29] Jacquin MF, Rhoades RW, Enfiejian HL, Egger MD (1983). Organization and morphology of masticatory neurons in the rat: a retrograde HRP study. *Journal of Comparative Neurology*.

[30] Marfurt CF (1981). The central projections of trigeminal primary afferent neurons in the cat as determined by the transganglionic transport of horseradish peroxidase. *Journal of Comparative Neurology*.

[31] Di Lazzaro V, Quartaroue A, Higuchi K, Rothwell JC (1995). Short-latency trigemino-cervical reflexes in man. *Experimental Brain Research*.

[32] Mannheimer JS, Rosenthal RM (1991). Acute and chronic postural abnormalities as related to craniofacial pain and temporomandibular disorders. *Dental Clinics of North America*.

[33] Krauss SL, Kraus SL (1994). Cervical spine influences on the management of TMD. *Temporomandibular Disorders*.

[34] Cailliet R (1979). *Soft Tissue Pain and Disability*.

[35] Darling DW, Kraus S, Glasheen-Wray MB (1984). Relationship of head posture and the rest position of the mandible. *The Journal of Prosthetic Dentistry*.

[36] Eriksson P-O, Häggman-Henrikson B, Nordh E, Zafar H (2000). Co-ordinated mandibular and head-neck movements during rhythmic jaw activities in man. *Journal of Dental Research*.

[37] Check P, Curl D, Curl DD (1994). Posture and craniofacial pain. *Chiropractic Approach to Head Pain*.

[38] Gregory TM (1993). Temporomandibular disorder associated with sacroiliac sprain. *Journal of Manipulative and Physiological Therapeutics*.

[39] Klasser GD, Greene CS (2009). Oral appliances in the management of temporomandibular disorders. *Oral Surgery, Oral Medicine, Oral Pathology, Oral Radiology and Endodontology*.

[40] Jackson BL, Barker W, Bentz J, Gambale AG (1987). Inter- and intra-examiner reliability of the upper cervical X-ray marking system: a second look. *Journal of Manipulative and Physiological Therapeutics*.

[41] Owens EF (1992). Line drawing analyses of static cervical X ray used in chiropractic. *Journal of Manipulative and Physiological Therapeutics*.

[42] Sigler DC, Howe JW (1985). Inter- and intra-examiner reliability of the upper cervical X-ray marking system. *Journal of Manipulative and Physiological Therapeutics*.

[43] Kowalski HM, Cohen WA, Cooper P, Wisoff JH (1987). Pitfalls in the CT diagnosis of atlantoaxial rotary subluxation. *American Journal of Roentgenology*.

[44] Lukhele M (1996). Atlanto-axial rotatory fixation. *South African Medical Journal*.

[45] Fielding JW, Stillwell WT, Chynn KY, Spyropoulos EC (1978). Use of computed tomography for the diagnosis of atlanto-axial rotatory fixation. A case report. *Journal of Bone and Joint Surgery. American*.

[46] Sessle BJ (1999). The neural basis of temporomandibular joint and masticatory muscle pain. *Journal of Orofacial Pain*.

[47] American Academy of Orofacial Pain (1996). *Guidelines for Assessment, Diagnosis, and Management*.

[48] Shankland WE (2001). Migraine and tension-type headache reduction through pericranial muscular suppression: a preliminary report. *Cranio*.

[49] Farrar WB (1972). Differentiation of temporomandibular joint dysfunction to simplify treatment. *The Journal of Prosthetic Dentistry*.

[50] Cooper BC (1997). The role of bioelectronic instrumentation in the documentation and management of temporomandibular disorders. *Oral Surgery, Oral Medicine, Oral Pathology, Oral Radiology, and Endodontics*.

[51] Gelb ML, Gelb H (1991). Gelb appliance: mandibular orthopedic repositioning therapy. *Cranio Clinics International*.

[52] Lerman MD (1974). The hydrostatic appliance: a new approach to treatment of the TMJ pain-dysfunction syndrome. *The Journal of the American Dental Association*.

[53] Lee YJ (2007). *Cerebrospinal Functional Medicine*.

[54] Yin CS, Lee YJ (2011). FCST, its foundation and early history. *Jorunal of TMJ Balancing Medicine*.

[55] Shon IC, Ahn KS, Sohn KS (2006). Two cases of spasmodic torticollis managed by yinyang balance appliance of FCST for the meridian and neurologic balance. *The Korean Journal of Maridian & Acupoint*.

